# A Case Report Exploring Early-Onset Alzheimer’s Disease With No Known Family History

**DOI:** 10.7759/cureus.90576

**Published:** 2025-08-20

**Authors:** Jasmine Bains, Omotola Ogunjobi

**Affiliations:** 1 Medicine, The Queen Elizabeth Hospital, Birmingham, GBR; 2 General Adult Psychiatry, Black Country Healthcare NHS Foundation Trust, Birmingham, GBR

**Keywords:** alzheimer’s dementia, alzheimer's disease and epilepsy, atypical alzheimer's dementia, behavioral and psychological symptoms of dementia (bpsd), cognitive impairment and dementia, dementia patients, early-onset dementia, neuro-imaging, neuroimaging and dementia, young-onset dementia

## Abstract

This case report conveys a rare presentation of early-onset Alzheimer's disease (EOAD) in a 58-year-old female with no family history of dementia. The patient demonstrates progressive cognitive decline with features of memory loss and associated severe language impairment, subsequently leading to a delay in the definite diagnosis. A systematic literature review using PubMed and Google Scholar was undertaken to explore the features of EOAD and how it differs from late-onset Alzheimer's disease (AD), focusing on risk factors, clinical presentation, investigation, and management. Although this patient was not known to have a family history of AD, evidence suggestive of EOAD could be observed within a series of neuroimaging and diagnostic tests such as magnetic resonance imaging scans, cerebrospinal fluid analysis, and blood tests. Management of EOAD involves a combination of pharmacological and non-pharmacological interventions using a biopsychosocial approach. Overall, the case highlights challenges in recognition of EOAD in its early stages of presentation for patients with no family predisposition, whilst emphasizing the importance of early detection and management to help improve patient outcomes. It also focuses attention on the significant impact a condition such as EOAD can have on the family members involved. Further research would be required to potentially redefine diagnostic criteria and treatment strategies for conditions of such rarity.

## Introduction

Alzheimer's disease (AD) is a progressive neurodegenerative disorder that commonly presents with progressive cognitive decline, impaired memory, and noticeable behavioral change [1]. Early-onset AD (EOAD) describes a cohort of patients under the age of 65 years where these features can be observed, approximately affecting 5%-6% of patients with AD [1]. EOAD differs significantly in comparison to late-onset AD (LOAD) in terms of clinical presentation, pathophysiology, and overall progression of the disease. EOAD can involve executive dysfunction and language impairment with a closer correlation toward genetics; however, both environmental and lifestyle factors can play a key role [2]. EOAD is frequently associated with autosomal dominant mutations in three genes: Amyloid precursor protein (*APP*), presenilin 1 (*PSEN1*), and presenilin 2 (*PSEN2*) [3]. Diagnosis of EOAD can be challenging, particularly when there are variants of the presentation, as demonstrated within the case. Throughout the case presentation, we examine the different phases the patient went through, from struggling with the expression of speech to a rapid decline in cognition, as well as misdiagnoses at different stages of her presentation. She went through a multitude of investigations, including neurophysiology, blood tests, and a thorough review by multiple medical specialties to help ascertain an explanation for her symptoms. Despite the absence of known genetic predisposition, a diagnosis of EOAD was made following multiple years of ambiguity, which highlights the challenges of diagnosing EOAD with current standards of investigations. This case report aims to explore the clinical presentation, diagnosis, and management of EOAD, suggesting the importance of early detection and how this can have a significant impact on both the patient and family members involved.

Method

A comprehensive case report was conducted to explore EOAD, focusing on its distinct characteristics in comparison to LOAD. A systematic literature review was conducted using PubMed and Google Scholar to identify relevant studies and case reports. Keywords used within the search included "early-onset Alzheimer’s disease", "genetic factors in early-onset Alzheimer’s disease", "investigations in EOAD", "diagnosis in EOAD", and "management of EOAD". The review aims to explore the potential risk factors, investigations, and clinical management of EOAD and how early detection can have a significant impact on the quality of life of the patient.

## Case presentation

Background

The case presents a 58-year-old female who was born in the UK. She was a full-term baby with no notable complications during her early stages of life in terms of education and work life. She previously lived independently, working as a hospital cleaner, until her cognitive decline affected her daily functioning. Before her diagnosis of EOAD, her medical history included hypertension, hypothyroidism, epilepsy, vitamin B12 deficiency secondary to pernicious anemia, chronic constipation, recurrent urinary tract infections (UTIs), and a possible multiple transient ischemic attacks due to observations of facial asymmetry. She was diagnosed with depression and anxiety in 2017, which involved treatment with antidepressant medication. The patient has one son and two daughters and is a grandmother to two grandchildren. There is no history of substance abuse, having previously only consumed alcohol during social occasions, and now no longer drinks alcohol. Additionally, there is no forensic history to date.

History of presentation

In December 2019, the patient started to experience progressive cognitive decline with severe language impairment, where she described a great limitation in communicating verbally. At this time, she could still retain and understand simple English; however, she struggled with complex conversations. This was observed to be the trigger of her anxiety in the initial stages, as she felt people were noticing her difficulties with speech. She was referred to the community mental health team (CMHT) for further investigations, where standardized tools such as Addenbrooke’s Cognitive Examination-Revised (ACE-R) test and Patient Health Questionnaire-9 (PHQ-9) were used. Here, she scored 2 (medium risk) on the PHQ-9 scale for depression and scored 53/100 in the ACE-R, suggesting likely cognitive impairment. A mini-mental state examination conducted in early 2020 showed she was having excessive periods of sleep and maintained a good appetite with no suicidal thoughts, hallucinations, or delusions. Her family stated she was becoming more forgetful, eventually concluding she was no longer considered safe to go outside without support. During this period, she was commenced on fluvoxamine 100 mg at night and 75 mg of aspirin. Further follow-up included blood tests, a memory clinic assessment, and an outpatient MRI scan. Her blood tests were conducted (results shown below in Table 1), which were reviewed by the memory assessment service (MAS) in 2020. As you can see below, the blood tests indicated low vitamin B12 and folate, suggesting a combined deficiency. Elevated thyroid-stimulating hormone with low levels of thyroxine may indicate hypothyroidism contributing to cognitive symptoms. The MAS team suggested her cognitive decline was possibly due to reversible causes such as vitamin B12 deficiency; therefore, she was discharged from MAS and commenced on vitamin B12 replacement.

**Table 1:** Blood tests conducted in November 2020 T4: Thyroxine; TSH: Thyroid-stimulating hormone

Parameters	Patient values	Reference range
Serum free T4 (pmol/L)	9.5	12.0-22.0
Serum TSH (miU/L)	9.5	0.27-4.2
Serum ferritin (ug/L)	23.0	<15.0
B12 (ng/mL)	109	180-1000
Folate (pg/mL)	3.0	>4.0

The patient was further followed up with a telephone consultation in July 2021 with CMHT, where she received a primary diagnosis of F067 mild cognitive disorder with a secondary diagnosis of Z004 (general psychiatric examination not elsewhere classified). During this period, collateral history from her family suggested worsening of symptoms such as being unable to go out alone, struggling with word finding, and being very low in mood, which can quickly escalate to anger and aggression. She appeared to be paranoid on occasions but had no signs of hallucinations. At this point, there were suggestions of possible mild cognitive impairment secondary to a vascular cause; however, further investigations were needed for clarification. She commenced on 50 mg sertraline in the morning with her general practitioner, which was increased to 100 mg in the morning after three weeks. She was due to have further follow-up in January 2022 with the CMHT team; however, due to disengagement with the services, she was discharged.

Later in the year, she was admitted to the hospital due to repeated seizure episodes, with a background of three-year cognitive impairment. She was assessed by the liaison psychiatry team once inflammatory causes were ruled out by the medical team. They described the patient as being “confused and anxious,” often responding with “I don’t know,” with noticeable expressive dysphasia. Given the long duration of her clinical symptoms, she was discussed with the neurology consultant, who suggested an outpatient cerebrospinal fluid (CSF) analysis and ambulatory electroencephalography (EEG) for further assessment. She was discussed in the neuroradiology multidisciplinary team meeting, where the first suspicions of EOAD were raised. Further CSF samples suggested the likelihood of Alzheimer’s dementia, and she was referred to the Rare Dementia Service. Although there is no known family history of AD, it was recommended to have a referral to the clinical genetics team for genetic assessment to recognize if genetics will be a risk factor for her children. Genetic testing confirmed no pathogenic mutations in the *APP*, *PSEN1*, or *PSEN2* genes. A summary of the investigations throughout her presentation can be seen in Table 2, which showcases the progression of her disease throughout the years. Serial MRI scans showed a significant decrease in global brain volume between June 2020 and September 2022, correlating with worsening cognition.

**Table 2:** Timeline of investigations throughout the early stages of disease progression AD: Alzheimer’s disease; CSF: Cerebrospinal fluid; FLAIR: Fluid-attenuated inversion recovery

Date	Investigation
June 2020	MRI head (Figure 1): "Brain volume appears to be relatively well maintained with no significant cerebral, cerebellar, or hippocampal volume loss identified. Scattered non-specific T2/FLAIR hyperintensities are observed in the supratentorial white matter. No evidence of acute or chronic infarction. No intracranial hemorrhage, mass lesion, or subdural collection. No midline shift or hydrocephalus. The midline structures and craniocervical junction appear unremarkable. Satisfactory appearances of the vascular flow voids."
November 2021	MRI head (Figure 2): "Involutional brain changes observed with prominent ventricles, CSF systems, and cortical sulci. Bilateral periventricular white matter foci of high T2 signal intensity, observed with no restricted diffusion, are likely ischemic. No intra- or extra-axial space-occupying lesion, hemorrhage, or gross territorial infarct. Normal appearance of the midline structures and the visualized craniocervical junction. No evidence of brain herniation or midline shift. Normal flow void of the intracranial vessels is observed."
September 2022	MRI head (Figure 3):"Slow progressive decrease in central and cortical cerebral volume, excessive for age when compared to scans dating back to June 2020. The volume loss is generalized with no preferential lobar atrophy. No cerebral microbleeds or surface hemosiderosis. No abnormal striatal or neocortical diffusion restriction. Non-specific T2/FLAIR hyperintensities scattered in the subcortical and periventricular white matter of both cerebral hemispheres and the brain stem are similar in distribution and burden. No new focus of signal abnormality suggestive of inflammation or demyelination. Normal flow voids are maintained in the main intracranial arterial pedicles. The major dural venous sinuses are patent."
September 2022	CSF findings: They showed features of AD with raised total tau, low CSF amyloid 1-42/40 ratio, and a raised CSF phosphorylated tau 181.

**Figure 1:**
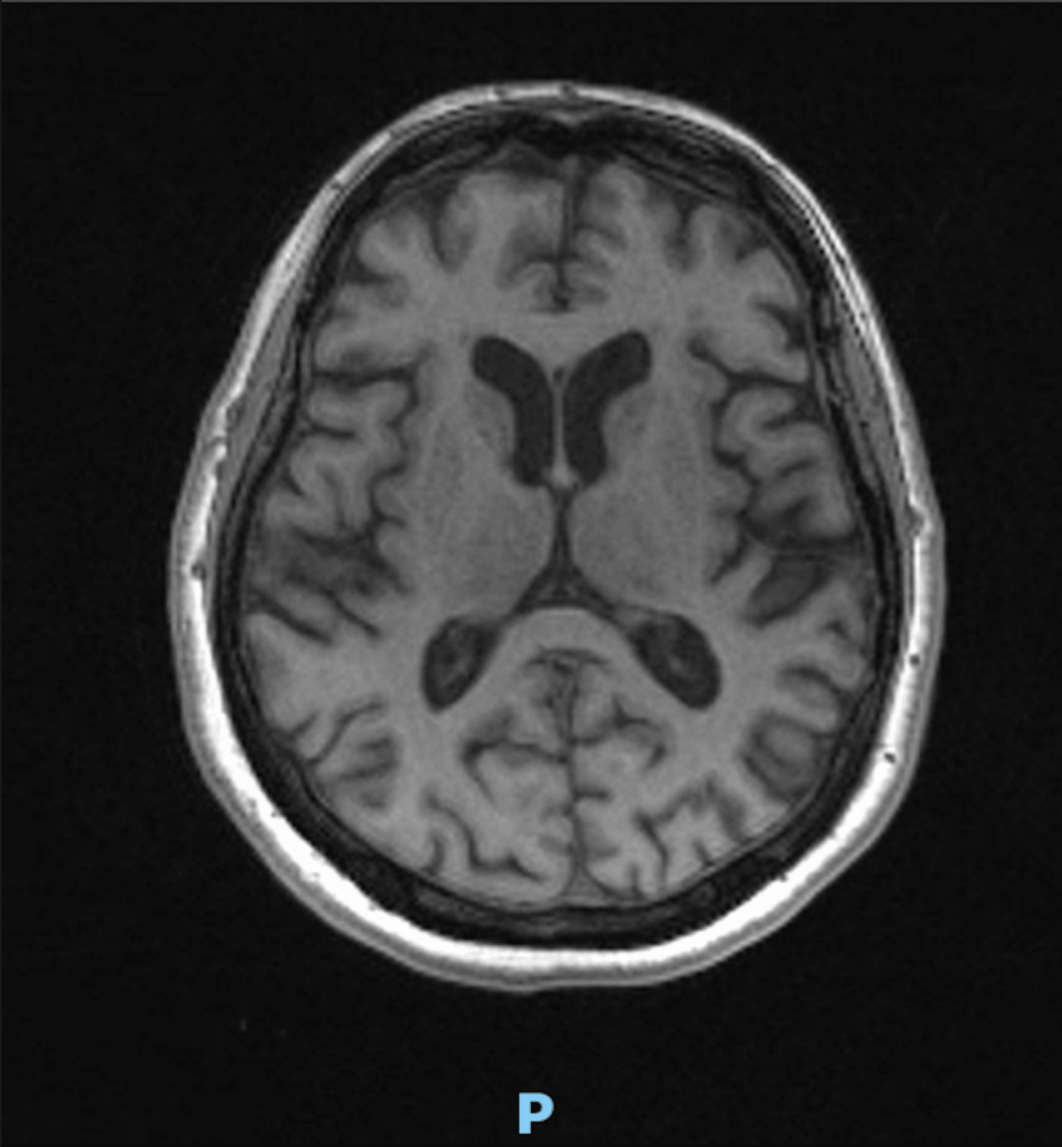
June 2020: MRI head showing possible mild chronic small vessel disease but no significant reduction in brain volume

**Figure 2:**
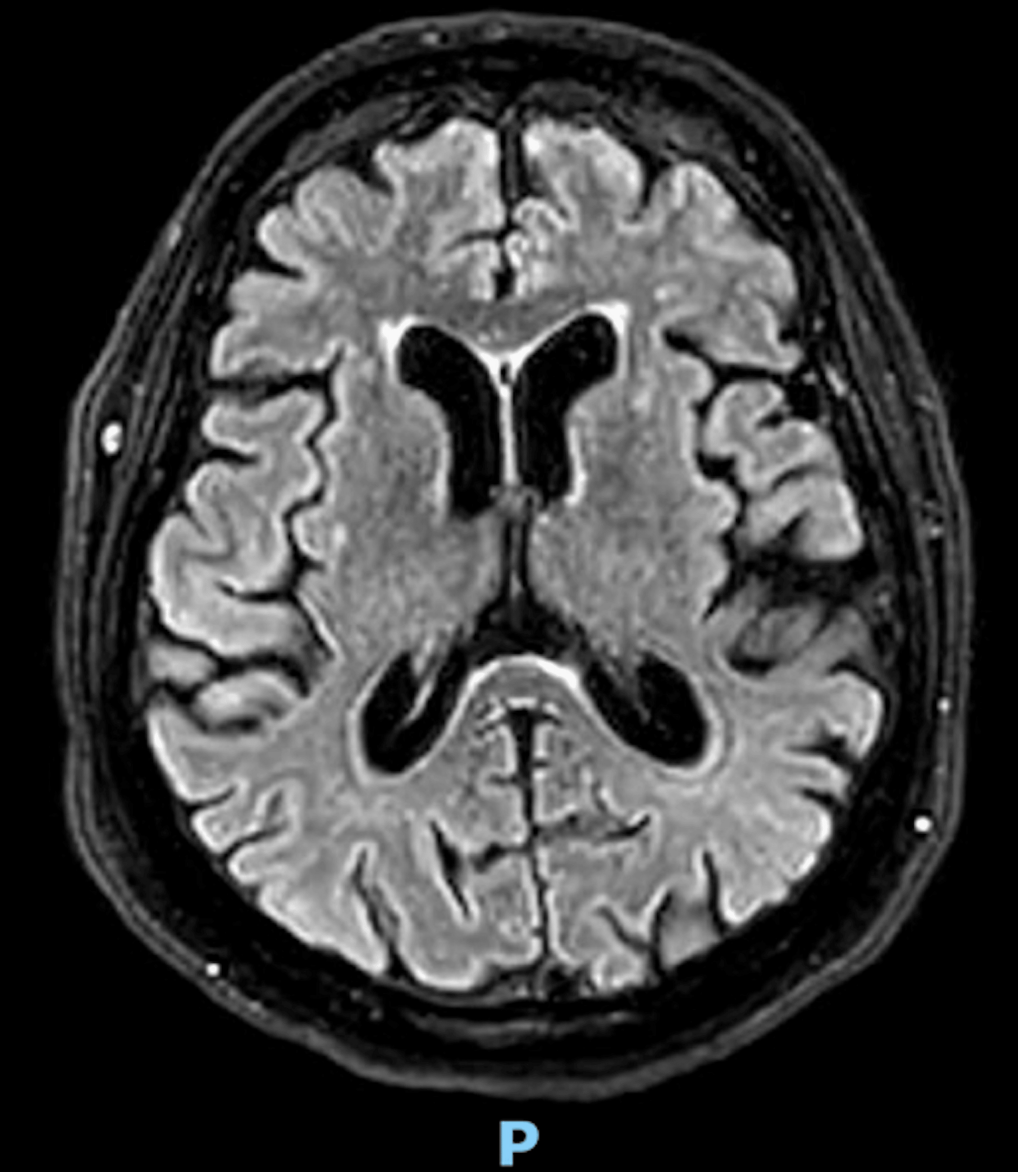
November 2021: MRI head showing mild small vessel disease, but no significant reduction in the size of the brain or any intracranial abnormalities

**Figure 3:**
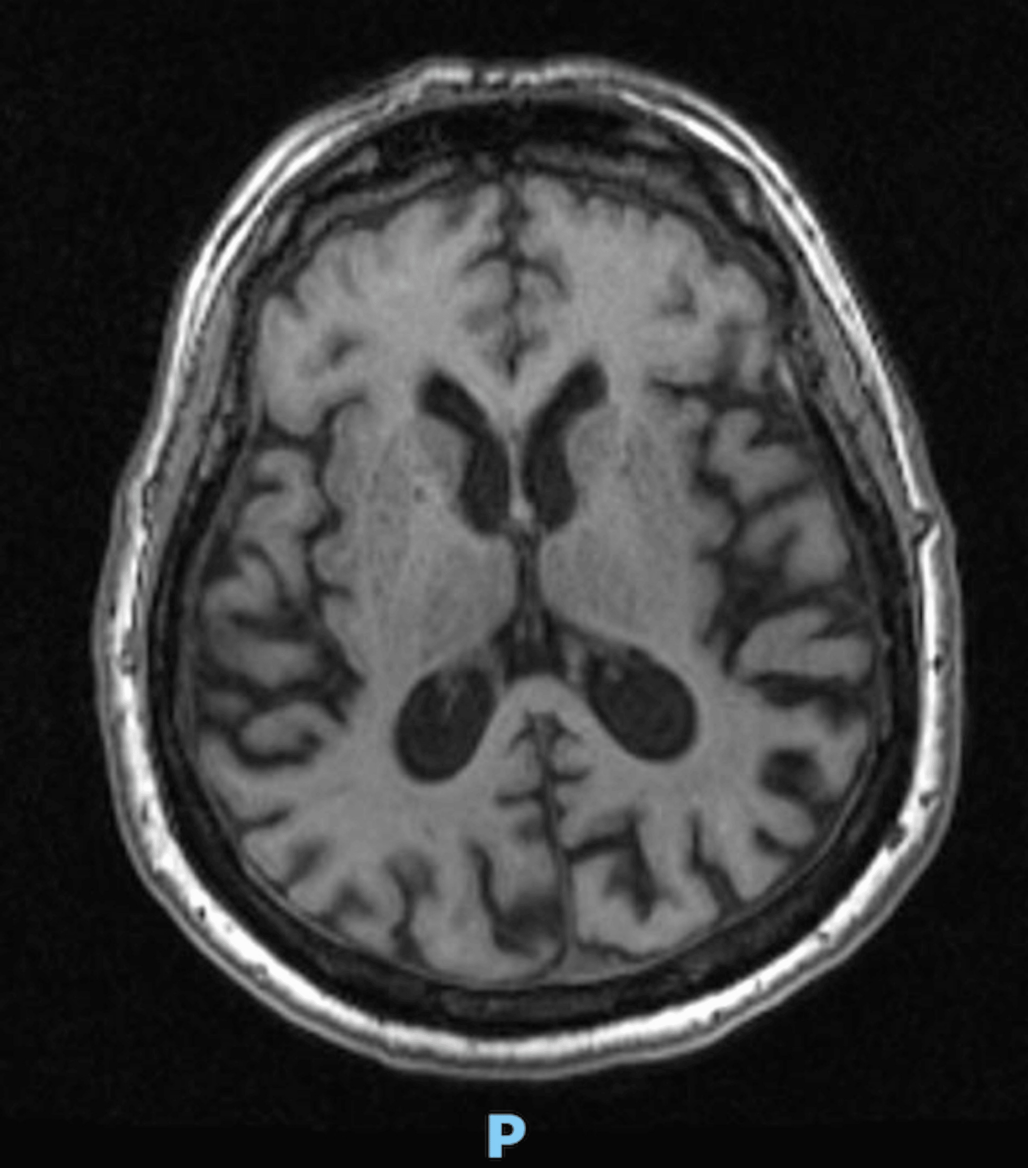
September 2022: MRI head showing progressive age, excessive reduction in global cerebral volume

In January 2023, she was assessed further by MAS, where her presentation suggested executive impairment, attentional and thinking speed impairment, and behavioral and personality changes. In addition, there were symptoms of organic affect, i.e., withdrawal, more changeable mood, restlessness, agitation, and overactivity. In August 2023, her medication was altered, swapping sertraline for mirtazapine 15 mg at night. She was also referred to speech and language therapies. A few months later, she commenced on donepezil 5 mg once daily (OD). Throughout the next few months, she experienced worsening hallucinations, disruption to sleep, an increase in physical and verbal abuse, and constant escape-seeking behavior, suggesting ineffective management with her current treatment. She was then trialed on alternative medication, which includes risperidone 0.5 mg twice daily (BD), rivastigmine 1.5 mg BD, zopiclone 7.5 mg BD, and PRN lorazepam 1 mg BD. In September 2024, she was diagnosed with behavioral and psychological symptoms of dementia, which included symptoms of acute anxiety, physical aggression, and damage to physical surroundings. In terms of physical aggression, the family was concerned, as this could be directed toward her grandchildren, including throwing objects, hitting, and kicking family members. Her behaviors had no specific trigger; however, they escalated to the point where the family began developing concerns for their own welfare and the welfare of the patient. She had developed exit-seeking behavior, being deemed a high risk to herself if left unsupervised on the street. Her family began to find it difficult to cope due to her increased agitation, and they were keen to receive support. She had lacked the capacity to provide consent for hospital admission; however, informal admission would not be appropriate given her presentation. She was assessed by the psychiatric team, who recommended that she be assessed and managed as an inpatient. The first medical recommendation was put in place, and she was admitted under section 2 of the Mental Health Act 1983.

Whilst an inpatient, she was treated using both pharmacological and non-pharmacological approaches. Pharmacological approach included initial treatment with risperidone 1 mg BD; however, this was not effective, therefore she was switched to haloperidol 0.5 mg BD, which had shown a reduction in her agitation and aggression toward others. Non-pharmacological strategies included redirection and gentle support, which were implemented on the ward and were to be continued at home after discharge. Additionally, she had dementia care mapping, which is a psychologist-led assessment, providing insights into her behavior and helping management of challenging behaviors more effectively. Lastly, other components of her physical health were cared for, such as epilepsy, chronic constipation, and monitoring for recurrent UTIs to prevent escalation in confusion and changes in behavior. Following stabilization of her dose of 0.5 mg BD haloperidol, mirtazepine 30 mg at night, lamotrigine 75 mg OD for her epilepsy, and non-pharmacological strategies, she was discharged home with community support services in place.

## Discussion

AD is classified as a neurodegenerative disorder due to extracellular amyloid-positive neuritic plaques and intracellular tau-positive neurofibrillary tangles of AD in the brain [1]. Within the late 1960s and 1970s, investigations reported a vast presence of similar neuritic plaques and neurofibrillary tangles in elderly patients with dementia [1]. This led to a primary focus on LOAD, which ultimately led to a reduction in research on EOAD, despite around 5%-6% of AD cases being found to have EOAD [1].

EOAD is defined as having an age of onset younger than 65 years, being one of the most common causes of early-onset neurodegenerative dementia. We can observe differences between phenotypic presentation in patients with EOAD and patients with LOAD, including non-amnestic phenotypic variants sparing the hippocampus and greater tau burden in the posterior neocortices [1]. EOAD has a genetic predisposition that involves autosomal dominant transmission and higher polygenic susceptibility, therefore, increasing the risk of development of AD among family members [2]. EOAD can be commonly accompanied by secondary dementias such as multiple sclerosis dementia, alcohol-related dementia, traumatic brain injury, and viral, immunological, and neoplastic conditions [4].

It is also important to clearly elaborate on the differences between early-onset and late-onset dementia based on genetics and clinical presentation to emphasize the complexities of EOAD and the need for the development of our understanding and awareness of such cases. Table 3 provides information about these differences [5].

**Table 3:** Key differences between EOAD and LOAD The table has been created by the author using the resource [5]. APOE: Apolipoprotein E; *APP*: Amyloid precursor protein; EOAD: Early-onset Alzheimer’s disease; LOAD: Late-onset Alzheimer’s disease; *PSEN1*: Presenilin 1; *PSEN2*: Presenilin 2

Feature	EOAD	LOAD
Main genes implicated	*APP*, *PSEN1*, and *PSEN2* in 10%-15% of cases	APOE (especially the E4 allele)
Pattern of inheritance	Autosomal dominant (familial)	Complex with numerous environmental factors
Effect of mutation	Direct causality by pricing abnormal amyloid-beta proteins	Increased risk of developing disease; not a 100% chance
Age of onset	Before the age of 65 years	Usually, after the age of 65 years
First set of symptoms	Difficulties with language, executive functioning, and visuospatial orientation	Loss of memory is most distinct
Symptom progression	Rapid and severe deterioration	Insidious decline
Prevalence	5%-10% of all Alzheimer’s disease	90%-95%

Clinical features 

A diagnosis of EOAD requires a distinct approach that differs from current standards of investigations for LOAD. There is a wide range of mental, behavioral, cognitive, and neurological symptoms, which can present at different levels of severity. Those with autosomal dominant inheritance can experience atypical features such as headaches, seizures, pseudobulbar palsy, hyperreflexia, and myoclonus [6]. In the initial stages of the disorder, associated issues such as depression and menopause can be observed, which may not present as obvious symptoms of EOAD at first [4]. A more rapid deterioration of symptoms can be observed in patients with EOAD, increasing the risk of mortality by nearly double [2]. This is reported in a study focusing on elderly Korean patients with AD, where many premature deaths were observed in patients between the ages of 40 and 65 years diagnosed with EOAD in comparison to those who had no formal diagnosis of dementia [2]. They were also found to experience a greater functional impairment in comparison to LOAD. Despite this, there is a decreased risk of cerebrovascular events, diabetes mellitus, and obesity in comparison to LOAD [2]. Furthermore, in a study conducted by Joubert [7], results show that both early- and late-onset AD have noticeable impairment of memory executive functions, language, praxis, and visuoconstructional ability compared to control groups. However, those with LOAD had a greater level of impairment of their semantic memory than patients with EOAD [7]. Those with EOAD were more affected in other cognitive domains such as executive function and visuocontructional abilities [7].

Investigations and diagnosis 

To differentiate AD from other neuropathologies, a mixture of blood tests, neuroimaging, CSF analyses, tissue biopsies, and neurophysiology tests is used [8]. Many patients may be misdiagnosed with alternative conditions, such as depression, without further investigation, when this could suggest impairment of the executive function of the frontal lobe secondary to EOAD [4]. Here, the importance of both collateral and clinical history taking is essential to narrow differential diagnoses. Questions asked in history should focus on cognitive function, alcohol/substance abuse, family history, changes in premorbid personality, and history of both progressive neurological and chronic systemic disease [4]. Cognitive impairment would need to be assessed using neurophysiological testing and further bedside assessment of cognitive processes using tools such as the Montreal Cognitive Assessment or Mini-Mental State Examination [4]. Blood tests are required to diagnose autoimmune diseases, immunodeficiency viruses such as HIV, and toxic or metabolic causes [8]. Neuroimaging is now increasingly used, with a preference for MRI, where sequences can show brain atrophy patterns assisting in narrowing down differential diagnosis [4]. In EOAD, a greater widespread cortical atrophy in the parietal cortex can be observed in comparison with LOAD, which is usually limited to the temporal lobe [1]. In addition to this, EOAD shows a larger sulcal width in the temporoparietal cortex, a reduced amount of volume loss in the hippocampus, and noticeable atrophy of the posterior cingulate cortex. Overall, in EOAD, there are smaller functional changes in the cortical regions connected to the hippocampus in comparison to LOAD [1]. CSF analysis can be used to identify inflammatory or viral causes of memory loss and can be used in conjunction with MRI scans [4]. CSF total tau (t-tau) and phosphorylated tau (p-tau) are believed to reflect axonal neurodegeneration and tangle pathology, associated with an increased rate of atrophy for those with AD [9]. Within the CSF analysis of patients with EOAD, typically, a low amyloid β_1-42_ is observed, with high t-tau and p-tau levels [10].

Further neurophysiology tests include electromyography, nerve conduction studies, and EEG, which can help show myopathy, neuropathy, and seizure activity [4]. There have been recent developments in the use of fluorodeoxyglucose PET, which can show greater parietal hypometabolism in patients with EOAD, whereas in LOAD, a greater bilateral temporal hypometabolism is observed [1]. Amyloid PET scans can be useful in reducing differential diagnosis of patients presenting with suspected EOAD, as amyloid activity correlates with low CSF and amyloid-β8. Tau PET signals can better correlate with clinical symptoms, as a greater uptake is observed within the prefrontal and premotor regions and the inferior parietal cortex, for those with EOAD in comparison to LOAD [11]. In LOAD, higher uptake is confined to the regions of the temporal lobe [11]. A summary of the investigations used to aid the diagnosis of EOAD is shown below in Table 4.

**Table 4:** Different investigations used to aid diagnosis of EOAD The table has been created by the author using different resources as referenced within the table. EOAD: Early-onset Alzheimer's disease

Investigation	How it aids diagnosis
Blood tests	To look for reversible causes of reduced memory. To diagnose autoimmune diseases, immunodeficiency viruses such as HIV, and toxic or metabolic causes [6]. For example, full blood count, C-reactive protein, urea and electrolytes, thyroid function tests, serum B21 and folate levels
CT head	To visualize possible brain abnormalities, i.e., brain tumors and strokes, which could be alternative causes of cognitive decline [6]. In dementia, atrophy of the brain is commonly observed. In comparison to MRI, it is much more cost-effective and readily available; however, it cannot be used for definitive diagnosis.
MRI head	To visualize the brain structure and identify potential abnormalities, which could cause cognitive decline, such as brain tumors, vascular lesions, etc. [6]. In dementia, atrophy of the brain is commonly observed. Allows us to differentiate between the types of dementia. Allows monitoring of disease progression
Cerebrospinal fluid analysis	To rule out infective cause such as bacterial/viral/fungal. To measure amyloid β_1-42_, total tau, and phosphorylated tau proteins [8]
Fluorodeoxyglucose PET	Amyloid PET scans can be useful in reducing differential diagnosis of patients presenting with suspected EOAD [9]. Tau PET signals can better correlate with clinical symptoms.
Electromyography	To rule out myopathy [6]
Nerve conduction studies	To rule out neuropathy [6]
Electroencephalography	To rule out seizures [6]

Management 

Effective early intervention has been shown to slow the progression of cognitive decline. The management of EOAD is similar to that of LOAD, with a particular focus on specific cognitive areas and more age-appropriate psychosocial support and education [1]. This includes the use of acetylcholinesterase inhibitors such as donepezil, galantamine, and rivastigmine, which primarily target memory [1]. 

These treatments can also be beneficial for those with logopenic variant primary progressive aphasia and other variants of EOAD such as posterior cortical atrophy [1]. When addressing specific cognitive and behavioral deficits, there may be differences between the management of EOAD and LOAD; for example, speech therapy assessment can be used to help focus on aphasia and improve communication [1]. Patients with behavioral and executive dysfunction may benefit from the use of psychoactive medications to help manage them [1]. A key consideration in the management of EOAD is the significant impact of the diagnosis on the patient and their family, emphasizing the need for comprehensive support.

## Conclusions

In conclusion, this case report highlights the complexities and challenges associated with diagnosing and managing EOAD in individuals with no known family history of dementia. The patient presented with a range of cognitive, behavioral, and psychological symptoms that required careful and comprehensive assessment and multidisciplinary management to reach a definitive diagnosis. Through a combination of neuroimaging, neurophysiological testing, and comprehensive history taking, a diagnosis of EOAD was identified, despite the absence of a genetic predisposition, showcasing the importance of prompt and thorough diagnostic workup in middle-aged patients presenting with progressive cognitive decline. The findings from the literature and case study emphasize the significant differences between EOAD and LOAD, particularly focusing on symptom presentation, progression, and neurobiological changes. Early detection and diagnosis are critical in improving the quality of life for patients, allowing a focused and personal approach to interventions and management strategies, including pharmacological treatments and psychosocial support. The case also highlights the significant impact that EOAD has on the patient’s family and the importance of providing family members involved with adequate support and education to cope with the challenges associated with caring for a patient with dementia. Overall, this case study contributes to the developing understanding of EOAD and emphasizes the need for increased awareness and clinical vigilance in diagnosing this condition. Further research is required to gain a better understanding of the pathophysiology, genetic factors, and optimal management strategies for EOAD, particularly for individuals without a known family history.
